# Dataset for *aedes aegypti* (diptera: Culicidae) and *culex quinquefasciatus* (diptera: Culicidae) collections from key West, Florida, USA, 2010–2020

**DOI:** 10.1016/j.dib.2022.107907

**Published:** 2022-02-02

**Authors:** Catherine A. Pruszynski

**Affiliations:** Florida Keys Mosquito Control District, 18 Aquamarine Drive, Key West, FL 33040, USA

**Keywords:** Dengue, Vector, Trapping, Mosquito, Surveillance

## Abstract

The Florida Keys Mosquito Control District began deploying Biogents® BG Sentinel traps to monitor *Aedes aegypti* (Diptera: Culicidae) populations in Key West during a small autochthonous dengue outbreak that began in November 2009. This paper provides weekly data for twelve collection points from January 2010 through December 2020. BG Sentinel traps were baited with dry ice and proprietary BG Lure and were set in the afternoon and retrieved the following morning totalling 19 collection hours. Trap collections also included *Culex quinquefasciatus* and thus data for that species is also included. The collection data could provide insight into dengue transmission in a small sub-tropical US city.

## Specifications Table

**Table:** 

Subject	Insect Science
Specific subject area	Entomology, seasonal distribution, relative abundance
Type of data	spreadsheet
How the data were acquired	Mosquito collections in Key West, FL, USA
Data format	Raw
Description of data collection	Dry-ice and BG Lure baited Biogents® BG Sentinel traps were deployed in the afternoon and retrieved the following morning allowing for a 19 hour collection period. Traps were set weekly at twelve collection points in Key West, FL, USA. Collections were identified by a trained mosquito identification specialist.
Data source location	• Key West, Florida • USA Collection Locations: • 916 Eisenhower (24.558339854675495, −81.78846122012531) • 710 Bakers Lane (24.555955182122755, −81.79868330594911) • West Cemetery (24.555194, −81.796436) • 803 Olivia (24.55469875240301, −81.79591833388965) • 706 Amelia (24.550955010973, −81.79580228229688) • 719 Chapman Lane (24.551671354594614, −81.80339289294875) • Gardens Hotel (24.554432269860705, −81.79993886605854) • 1121 Margaret (24.55263325529155, −81.79332218132761) • 1306 Catherine (24.55506922677007, −81.78830543290469) • 1218 Pearl (24.55527139796987, −81.78680946680599) • Indigenous Park (24.54884158796217, −81.78472044577472) • 2437 Harris Ave (24.56060502723454, −81.775192516621)
Data accessibility	With the article

## Value of the Data

• The data provide a 10 year sample of *Ae. aegypti* and *Cx. quinquefasciatus* collections in Key West
• The data may benefit those studying or modelling dengue transmission
• The data may be useful to those interested in understanding *Ae. aegypti* and *Cx. quinquifasciatus* population changes over time

## 1 Data Description

The data are in an Excel spreadsheet that contains 6862 rows with row 1 being the header row and row 2–6862 containing data. There are seven columns. Column A is the year of the collection, Column B is the week of the year the collection took place. Column C is the name of the collection point. Column D is titled “AAEG(f)” and contains the number of female *Ae. aegypti* collected from that location during the collection period. Column E is titled “AAEG(m)” and denotes the number of male *Ae. aegypti* collected. Column F is titled “CXQ(f)” and contains the number of *Cx. quinquifasciatus* females collected and column G is titled “CXQ(m)” and notes the number of male *Cx. quinquefasciatus* collected from that location during the collection period. Cells with ND contain no data for that collection point for that week.

## 2 Experimental Design, Materials and Methods

The spreadsheet contains data for weekly Biogents BG Sentinel trap collections from twelve locations in Key West from January 2010 to December 2020. The traps were each baited with three pounds of dry ice in a small 0.5 gallon drink cooler that allowed carbon dioxide to sublimate over the trap. BG Lure was used as an additional bait. Each trap was powered with a 12 V, 7aH sealed lead acid rechargeable battery. Traps were set in the afternoon and retrieved the following morning for a 19 hour collection period. Collection nets were returned to the lab and frozen. Mosquitoes were identified by a certified mosquito identification specialist. On occasions where traps were not set or when traps were collected but not running properly, data cells contain ‘ND’ in the collection columns. On some occasions traps were not set or collected not running. In the data set, these traps are noted with a ‘ND’ in the collection columns. There are three published articles that also include these data [1], [2], [3].

## Ethics Statement

The author complies with ethical standards involved in obtaining these original data. All mosquito control activities were conducted in compliance with Florida State Statute 388 and rule chapter 5E-13 of the Florida Administrative code allowing the power to perform work on both public and private lands. Private yards were entered only after oral consent was obtained from individual residents. No specific permits were required for the collection of mosquitoes for this study.

## CRediT authorship contribution statement

**Catherine A. Pruszynski:** Investigation, Conceptualization, Data curation, Writing – original draft, Supervision.

## Declaration of Competing Interest

The authors declare that they have no known competing financial interests or personal relationships that could have appeared to influence the work reported in this paper.

## Appendix Supplementary materials

Image, application 1
